# Supplementary material for: Linseed Silesia, Diverse Crops for Diverse Diets. New Solutions to Increase Dietary Lipids in Crop Species
Source: Foods. 2021 Nov 3;10(11):2675. doi: 10.3390/foods10112675 (PMC8623773; doi:10.3390/foods10112675)
Supplement: Supplementary file 1 [file foods-10-02675-s001.zip › foods-1430258-supplementary.pdf]

Table S1 The content of antioxidants in seeds depending on variety.

mg/gFW	Linola	Silesia	Szafir
Ferulic acid	0.603± 0.022	0,678± 0.025	0.626± 0.024
Ferulic acid glucoside	1.528± 0.026	1.704± 0.018	1.614± 0.017
Coumaric acid	0.366± 0.018	0.598± 0.023	0.527± 0.016
Coumaric acid glucoside	1.034± 0.041	1.028± 0.001	1.064± 0.025
Caffeic acid	0.066± 0.010	0.126± 0.081	0.089± 0.022
Caffeic acid glucoside	0.716± 0.009	0.934± 0.011	0.796± 0.010
Vitexin (apigenin glucoside)	0.28± 0.005	0.16± 0.002	0.25± 0.004
Proanthocyanidin	0.025±0.002	0.052± 0.008	0.31± 0.003
Hydrolysable tannins	0.048± 0.001	0.279± 0.013	0.157± 0.011
γ- Tocopherol	8.337± 0.610	8.416± 0.350	8.363± 0.07
Plastochromanol-8	1.148± 0.075	1.220± 0.067	1.321± 0.081
Lutein	0.046± 0.013	0.046± 0.007	0.039± 0.007
